# Ectopic pancreatic fat as a risk factor for hypertension in children and adolescents with nonalcoholic fatty liver disease

**DOI:** 10.1111/jch.14326

**Published:** 2021-07-16

**Authors:** Eun Hye Lee, Ji Young Kim, Hye Ran Yang

**Affiliations:** ^1^ Department of Pediatrics Nowon Eulji Medical center Eulji University School of Medicine Seoul South Korea; ^2^ Department of Radiology Seoul National University Bundang Hospital Seongnam South Korea; ^3^ Department of Pediatrics Seoul National University Bundang Hospital Seongnam South Korea; ^4^ Department of Pediatrics Seoul National University College of Medicine Seoul South Korea

**Keywords:** hypertension, metabolic syndrome, pathophysiology, pediatrics, risk assessment

## Abstract

Although nonalcoholic fatty liver disease (NAFLD) is known to be a risk factor for cardiovascular diseases, few studies have reported an association between ectopic fat deposition and metabolic complications, including hypertension, in children with NAFLD. The present study evaluated the risk factors for hypertension in children with NAFLD from the aspect of ectopic fat. This cross‐sectional retrospective study investigated 65 children with NAFLD (49 boys, mean age 13.0 ± 3.2 years, mean body mass index z‐score [BMI‐z] 2.5 ± 1.2), who underwent liver biopsy and magnetic resonance imaging‐based fat fraction measurement for ectopic hepatic and pancreatic fats, as well as anthropometry, blood pressure, laboratory tests, and body composition analysis. A logistic regression model was used to identify the risk factors for hypertension. Through a simple logistic regression analysis, age (OR 1.392), BMI‐z (OR 3.971), waist circumference‐to‐height ratio (OR 1.136), fat‐free mass index (OR 1.444), γ‐glutamyl transferase (OR 1.021), quantitative insulin sensitivity check index (OR 0.743), dyslipidemia (OR 5.357), and pancreatic fat fraction (PFF) (OR 1.205) were associated with hypertension. The optimal cut‐off of PFF to divide children with NAFLD into two groups with and without hypertension was 4.39% (area under the curve 0.754, *p* = .001, sensitivity 82.4%, specificity 73.9%). Multiple logistic regression analysis in the fully adjusted model revealed both BMI‐z (OR 4.912, 95% CI, 1.463–16.497) and PFF (OR 1.279, 95% CI, 1.007–1.624) were independent risk factors for hypertension. In conclusions, in addition to BMI‐z, ectopic pancreatic fat is an important risk factor for hypertension in children with NAFLD.

## 1 INTRODUCTION

The prevalence of childhood obesity is rapidly increasing worldwide. Correspondingly, obesity‐induced metabolic complications, including nonalcoholic fatty liver disease (NAFLD), cardiovascular diseases (CVD), type 2 diabetes mellitus (DM), and metabolic syndrome (MetS) have also increased.^1^, ^2^ In developed countries, NAFLD affects 5.5%–10.3% of all children and 27.8%–41.2% of children with obesity.^3^ NAFLD has been suggested as a strong cardiovascular risk factor closely related to insulin resistance (IR) and visceral fat.^4^, ^5^

Mounting evidence highlights that NAFLD is an independent risk factor for CVD in adults. However, to date, this association has not been fully substantiated in children with NAFLD.^6^ Recently several studies on children with NAFLD suggest that in children, as well as in adults, NAFLD might be associated with potential cardiovascular complications.^6^ This is independent of the coexistence of well‐known risk factors and features of MetS.^6^ The association between NAFLD and various aspects of CVD, including cardiac dysfunctions, atherosclerosis, and hypertension (HTN) in children has been investigated.^6^, ^7^, ^8^, ^9^ In particular, children with NAFLD are at an increased risk for HTN compared with obese children without NAFLD, a risk that persists over time.^8^, ^10^, ^11^

When circulating triglycerides (TG) and free fatty acid levels exceed the metabolic capacity through adipocyte hypertrophy and hyperplasia, ectopic fat accumulation in non‐adipose tissues such as the heart, liver, pancreas, and skeletal muscles in patients with obesity becomes an increasing risk.^12^, ^13^ This accumulation of dysfunctional visceral and ectopic fat causes low‐grade inflammation, oxidative stress, endothelial dysfunction, atherogenic dyslipidemia, and impaired glucose metabolism, ultimately leading to the development of CVD in patients with NAFLD.^14^, ^15^

Along with ectopic hepatic fat in NAFLD, pancreatic fat is also considered as an obesity‐induced ectopic fat depot, which may contribute to cardio‐metabolic disturbances such as NAFLD, HTN, DM, dyslipidemia, and MetS.^16^, ^17^ The pancreas is divided into pancreatic islets that secrete endocrine hormones such as insulin and glucagon and exocrine regions that secrete digestive enzymes.^18^ It is presumed that pancreatic fat accumulation might cause pancreatic islet inflammation and β‐cell dysfunction, resulting in deterioration of the insulin‐secreting capacity.^18^ However, the association between pancreatic steatosis and cardiovascular disturbances along with impaired glucose metabolism represented by β‐cell dysfunction and IR remains unclear.^12^, ^13^, ^16^ Furthermore, little is known about the association between magnetic resonance imaging (MRI)‐based ectopic fat accumulation in the liver and pancreas and metabolic components of obesity‐related complications including HTN, particularly in children with biopsy‐proven NAFLD.

Therefore, the present study aimed to investigate the risk factors associated with HTN in pediatric patients with NAFLD from the aspect of ectopic fat.

## 2 METHODS

### 2.1 Study patients

This retrospective cross‐sectional observational study included 65 children and adolescents with liver biopsy‐proven NAFLD who underwent both liver biopsy and abdominal MRI performed within 2 days from March 2014 through February 2020. All children underwent anthropometric measurements, blood pressure (BP), body composition, and laboratory evaluation tests. NAFLD was diagnosed as the presence of steatosis in ≥5% of hepatocytes in the absence of evidence of other causes of liver disease. Secondary etiologies of hepatic steatosis, systemic disease, and pancreatic disease were excluded.

In our study, six patients were ≥18 years of age. These six patients were enrolled in this study despite their relatively higher age because they had been followed up continuously in the “pediatric gastroenterology, hepatology, and nutrition center” of our hospital since they were diagnosed with NAFLD at an early age. In the 21^st^ edition of the Nelson textbook of Pediatrics, the definition of adolescence includes three groups based on age: early adolescence indicates the approximate age range of 10–13 years, middle adolescence is 14–17 years, and late adolescence is 18–21 years.^19^ Furthermore, the diagnostic criteria for elevated BP and HTN are the same in adolescents (≥13 years of age) and adults.^20^, ^21^ That for prediabetes and DM is the same in children/adolescents and adults.^22^ That for dyslipidemia encompassed patients from birth through 21 years.^23^ MetS was defined based on a revised version of the modified National Cholesterol Education Program Adult Treatment Panel III diagnostic criteria, which covered adolescents up to 19 years.^24^

The study conformed to the ethical guidelines of the Declaration of Helsinki (revised in Fortaleza, Brazil, October 2013) and the recommendations of the Ethics Committee of Seoul National University Bundang Hospital. This study was approved by the Institutional Review Board of Seoul National University Bundang Hospital (IRB No. B‐2103‐670‐105).

### 2.2 Anthropometric measurements and body composition measurements

Anthropometric parameters, including height, weight, and waist circumference (WC), were measured in all children using standardized methods; we calculated the waist circumference‐to‐height ratio (WHtR), body mass index (BMI), and BMI standard deviation score (z‐score). In the Korean national growth charts, WC z‐score (WC‐z) has not yet been developed; therefore, we used WHtR instead of WC‐z. Obesity was defined as BMI ≥95^th^ percentile and overweight as BMI between the 85^th^ and 95^th^ percentiles, adjusted for age and sex, according to the Korean National Growth Charts 2017.^25^ Central obesity was defined as WC ≥90^th^ percentile, adjusted for age and sex, according to the Korean National Growth Charts 2007.^26^

Body composition was measured using bioelectrical impedance analysis (InBody J10, Biospace Co., Ltd., Seoul, South Korea), and the total body fat mass and fat‐free mass were recorded. The fat mass index (FMI) and fat‐free mass index (FFMI) were calculated as the fat mass (kg) and fat‐free mass (kg), respectively, divided by the square of height (m^2^).

### 2.3 BP measurements

BP was measured at every hospital visit using a standard measurement method.^20^ Elevated BP was defined as for children aged < 13 years, SBP or DBP ≥ the 90^th^ percentile to < 95^th^ sex‐, age‐, and height‐specific percentile or SBP of 120–129 mm Hg and DBP < 80 mm Hg. Elevated BP for children aged ≥13 years was defined as SBP of 120–129 mm Hg and DBP < 80 mm Hg.^20^ Children with HTN were defined as SBP or DBP ≥95^th^ sex‐, age‐, and height‐specific percentiles or SBP ≥130 mm Hg or DBP ≥80 mm Hg for children aged < 13 years. HTN for children aged ≥13 years was defined as SBP ≥130 mm Hg or DBP ≥80 mm Hg.^20^

If the initial BP was elevated as described above, two additional auscultatory BP measurements were performed and averaged to define the BP category. Children whose BP readings corresponded to HTN on ≥3 occasions or BP remained elevated BP for 1 year or more underwent 24‐h ambulatory BP monitoring to confirm whether they actually had HTN.

### 2.4 Laboratory tests

Blood samples were obtained after a 10‐h overnight fast and included the following: total cholesterol (T‐chol), TG, low‐density lipoprotein cholesterol (LDL‐C), high‐density lipoprotein cholesterol (HDL‐C), aspartate aminotransferase (AST), alanine aminotransferase (ALT), total bilirubin, γ‐glutamyl transferase (GGT), uric acid, fasting plasma glucose (FPG), insulin, and glycated hemoglobin (HbA1c) levels.

The homeostasis model assessment of insulin resistance (HOMA‐IR) was calculated using the following formula: FPG (mg/dl) × fasting insulin (μU/ml)/405,^27^ and the quantitative insulin sensitivity check index (QUICKI) was calculated as follows: 1/log (HOMA‐IR × 405).^27^

Prediabetes and DM were defined based on the American Diabetes Association guidelines.^22^ Prediabetes was defined as having at least one of the following: FPG level of 100–125 mg/dl (impaired fasting glucose), 2‐h plasma glucose during oral glucose tolerance test of 140–199 mg/dl (impaired glucose tolerance), and an HbA1c value of 5.7%–6.4%.^22^ DM was defined as having at least one of the following: HbA1c value ≥6.5%, FPG ≥126 mg/dl, 2‐h plasma glucose ≥200 mg/dl during oral glucose tolerance test, and a random plasma glucose level ≥200 mg/dl in a patient with classic symptoms of hyperglycemia.^22^

Dyslipidemia was defined as having at least one of the following: T‐chol ≥200 mg/dl, LDL‐C ≥130 mg/dl, HDL‐C < 40 mg/dl, TG ≥100 mg/dl (age ≤9 years), or ≥130 mg/dl (age ≥10 years).^23^

MetS was diagnosed in children who met at least three of the following five criteria: TG ≥110 mg/dl, HDL‐C ≤40 mg/dl, BP ≥90^th^ age‐, sex‐, and height‐specific percentiles, WC ≥90^th^ sex‐specific percentile, and FPG ≥100 mg/dl^28^ revised from the previous criteria of FPG ≥110 mg/dl.^24^

### 2.5 Liver biopsy

Liver biopsy was performed by an experienced pediatric radiologist with ultrasound guidance, and the findings were interpreted by an expert liver pathologist who was blinded to the patients’ clinical data. All biopsy specimens were evaluated based on the NAFLD Clinical Research Network criteria, and the NAFLD activity score (NAS) was assessed.^29^ The degree of steatosis, lobular inflammation, portal inflammation, and hepatocyte ballooning (steatosis 0–3, lobular inflammation 0–3, portal inflammation 0–2, hepatocyte ballooning 0–2) were graded accordingly. Moreover, hepatic fibrosis was staged as 0–4. The NAS was calculated using an 8‐point scale as the sum of the scores for steatosis, lobular inflammation, and hepatocyte ballooning.

### 2.6 MRI‐based measurement of the hepatic and pancreatic fat fractions

Abdominal MRI examinations were performed using a 3.0 T MR scanner (Ingenia, Philips Healthcare, Best, The Netherlands). The modified DIXON‐Quant sequence was obtained in a single breath hold, which automatically reconstructed a proton density fat fraction (PDFF) map. We obtained the maps of water, fat, fat fraction, R2*, and T2* by post‐processing the acquired images using the software provided by the manufacturer. All data were transferred to the IntelliSpace Portal software (version 10.0; Philips, Amsterdam, The Netherlands). Selection of the region of interest (ROI) and fat fraction measurements were performed by an expert pediatric radiologist who was blinded to the patients’ clinical and histopathological data. Hepatic fat fraction (HFF) measurements were performed by drawing two different ROIs in the right and left hepatic lobes. MRI‐PDFF is known to have an excellent diagnostic value for the assessment of hepatic fat content in patients with NAFLD.^30^ HFF ≥5.0% on MRI‐PDFF is defined as a fatty liver because MRI‐PDFF is highly accurate compared to the histological steatosis.^30^ ROIs of pancreatic fat fraction (PFF) measurements were generated in the head, body, and tail of the pancreas. The normal range of PFF to define fatty pancreas has not yet been established.

### 2.7 Statistical analysis

Descriptive characteristics are presented as mean ± SD for normally distributed variables or medians and ranges for non‐normally distributed variables. Categorical measurements are expressed as absolute numbers and percentages. Intergroup differences were evaluated using an independent *t*‐test for parametric variables and the Mann‐Whitney *U*‐test for non‐parametric variables. Categorical variables were compared using chi‐square or Fisher's exact tests. Correlation between the continuous variables was tested using the Pearson's correlation matrix. Receiver operating characteristic (ROC) curves were constructed to examine the appropriate cut‐off for PFF and BMI z‐score (BMI‐z), respectively, to divide children with NAFLD into two groups, with and without HTN. Pairwise comparison of ROC curves was performed to confirm diagnostic superiority. A logistic regression model was used to identify the risk factors for HTN. Through a simple logistic regression analysis, we identified several variables associated with HTN. Multiple logistic regression analysis was performed in the fully adjusted model after selecting noncollinear covariates to predict the risk factors associated with HTN. A two‐sided *p* value of < .05, was considered statistically significant. All statistical analyses were performed using PASW Statistics software (version 25.0; SPSS Inc., Chicago, IL, USA) and MedCalc software (MedCalc 19.6.4, MedCalc software, Mariakerke, Belgium).

## 3 RESULTS

### 3.1 Patient characteristics

We recruited 65 children with NAFLD (49 boys, 16 girls, mean age 13.0 ± 3.2 years, range 5.6–19.3 years). Most children were overweight or obese (mean BMI‐z, 2.5 ± 1.2); 13 (20.0%) children were overweight, 47 (72.3%) were obese, and only five (7.7%) children were within the normal range (BMI < 85^th^ percentile) (Table 1). Central obesity was noted in 56 children (86.2%). We observed normal BP in 38 (58.5%), elevated BP in 10 (15.4%), and HTN in 17 (26.2%) children (Table 1). Prediabetes was diagnosed in 16 (24.6%) and DM in nine (13.8%) children (Table 1). Dyslipidemia was observed in 43 (66.2%) patients, and MetS was diagnosed in 27 (41.5%) children (Table 1).

**TABLE 1:** Demographic, anthropometric, laboratory, and magnetic resonance imaging based‐fat fraction data of 65 children with nonalcoholic fatty liver disease

Variable	Data
*Demographic, anthropometric, body composition parameter*	
Sex [boys : girls] (n, %)	49 (75.4%) : 16 (24.6%)
Age (year)	13.0 ± 3.2
Height (cm)	156.9 ± 15.2
Height z‐score	0.86 (‐3.40–2.82)
Weight (kg)	67.8 (36.4–130.9)
Weight z‐score	2.27 (‐2.80–5.01)
Waist circumference (cm)	92.6 ± 12.1
Waist circumference‐to‐height ratio	0.59 ± 0.06
Central obesity (*n*, %)	56 (86.2%)
BMI (kg/m^2^)	27.8 ± 4.6
BMI z‐score	2.5 ± 1.2
BMI group [normal BMI : overweight : obesity] (*n*, %)	5 (7.7%) : 13 (20.0%) : 47 (72.3%)
Total body fat mass (%)	35.8 (22.3–67.2)
Fat‐free mass (%)	64.1 (32.8–77.7)
Fat mass index (fat mass [kg] / height [m]^2^)	10.09 ± 3.03
Fat free mass index (fat free mass [kg] / height [m]^2^)	17.39 ± 3.01
Mean systolic BP (mm Hg)	118.0 (100–159)
Mean diastolic BP (mm Hg)	64.0 (52–90)
BP category [normal BP : Elevated BP : HTN] (*n*, %)	38 (58.5%) : 10 (15.4%) : 17 (26.2%)
*Laboratory parameter*	
AST (IU/L)	53.0 (17–226)
ALT (IU/L)	121.0 (20–366)
Bilirubin, total (mg/dl)	0.6 (0.2–1.3)
GGT (IU/L)	38.0 (12–184)
Uric acid (mg/dl)	6.5 ± 1.8
Total cholesterol (mg/dl)	184.3 ± 37.5
Triglyceride (mg/dl)	121.0 (48–364)
HDL‐C (mg/dl)	45.9 ± 7.7
LDL‐C (mg/dl)	110.3 ± 26.9
Dyslipidemia (n, %)	43 (66.2%)
Fasting plasma glucose (mg/dl)	94.0 (71–270)
Fasting insulin (mIU/L)	20.9 (4.6–75.6)
HbA1c (%)	5.4 (5.0–13.5)
HOMA‐IR	5.1 (1.0–17.0)
QUICKI	0.30 (0.26–0.39)
Diabetes category [normal : prediabetes : DM] (*n*, %)	40 (61.5%) : 16 (24.6%) : 9 (13.8%)
Metabolic syndrome (*n*, %)	27 (41.5%)
*Magnetic resonance imaging‐based fat fraction*	
Hepatic fat fraction (%)	24.3 (4.2–49.9)
Pancreatic fat fraction (%)	3.8 (0.4–26.9)

*Note*: Values are presented as mean ± standard deviations or median (range) or numbers (%).

*Abbreviations*: ALT, alanine aminotransferase; AST, aspartate aminotransferase; BMI, body mass index; BP, blood pressure; DM, diabetes mellitus.; GGT, γ‐glutamyl transferase; HbA1c, glycated hemoglobin; HDL‐C, high density lipoprotein cholesterol; HOMA‐IR, homeostatic model assessment of insulin resistance; HTN, hypertension; LDL‐C, low density lipoprotein cholesterol; QUICKI, quantitative insulin‐sensitivity check index.

### 3.2 Association between metabolic components and MRI‐measured ectopic fat fraction of the liver and pancreas

As for ectopic fat on MRI, the median HFF was 24.3% (range, 4.2%–49.9%) and the median PFF was 3.8% (range, 0.4%–26.9%). HFF was not significantly different according to the status of obesity, HTN, central obesity, dyslipidemia, prediabetes/DM, and MetS status. Moreover, PFF did not differ according to obesity, central obesity, dyslipidemia, prediabetes/DM, and MetS status. However, PFF was significantly different according to HTN status. The median PFF of the HTN group was 6.7% (range 1.0%–26.9%) and that of the non‐HTN group was 3.2% (range 0.4%–15.8%) (*p* = .002).

Both HFF and PFF did not correlate with BMI‐z (between HFF and BMI‐z, Pearson's correlation coefficient (*r*) = 0.125, *p* = .327 and between PFF and BMI‐z, *r* = 0.143, *p* = .264). Furthermore, HFF was not significantly correlated with PFF (*r* = 0.178, *p* = .162).

### 3.3 Simple logistic regression analysis for hypertension in children with NAFLD

Through a simple logistic regression analysis for HTN, the following parameters were considered as risk factors for HTN in children with NAFLD: age (odds ratio [OR] 1.392, *p* = .003), BMI‐z (OR 3.971, *p* < .001), WHtR (OR for HTN by every 0.01 increment of WHtR was 1.136, *p* = .024), FMI (OR 1.405, *p* = .003), FFMI (OR 1.444, *p* = .002), GGT (OR 1.021, *p* = .016), dyslipidemia state (OR 5.357, *p* = .038), QUICKI (OR for HTN by every 0.01 increment of QUICKI was 0.743, *p* = .036), MetS (OR 12.564, *p* < .001) and PFF (OR 1.205, *p* = .014) (Table 2).

**TABLE 2:** Simple logistic regression analysis for predicting risk factors associated with hypertension in 65 children with non‐alcoholic fatty liver disease

	Hypertension		
	OR	95% CI	*p*‐value
Age (year)	1.392	1.123–1.726	**.003**
Boys	3.008	0.623–15.316	.168
BMI z‐score	3.971	1.842–8.563	**<.001**
Obesity	2.172	1.069–72.018	**.043**
Waist circumference (cm)	1.124	1.056–1.197	**<.001**
WHtR	1.136	1.017–1.269	**.024**
Central obesity	3.200	0.370–27.697	.291
Total body fat (%)	1.055	0.978–1.138	.170
Fat‐free mass (%)	0.948	0.879–1.023	.169
FMI (fat mass/height[m]^2^)	1.405	1.124–1.756	**.003**
FFMI (fat free mass/ height[m]^2^)	1.444	1.141–1.828	**.002**
AST (IU/L)	1.003	0.992–1.014	.574
ALT (IU/L)	1.001	0.995–1.008	.671
Total bilirubin (mg/dl)	1.938	0.233–16.137	.541
GGT (IU/L)	1.021	1.004–1.038	**.016**
Uric acid (mg/dl)	1.333	0.969–1.833	.077
Total cholesterol (mg/dl)	1.007	0.992–1.022	.382
Triglyceride (mg/dl)	1.007	0.999–1.014	.089
HDL‐C (mg/dl)	1.005	0.935–1.080	.894
LDL‐C (mg/dl)	1.012	0.991–1.034	.260
Dyslipidemia	5.357	1.100–26.089	**.038**
Fasting plasma glucose (mg/dl)	1.018	0.998–1.038	.079
Insulin (mIU/L)	1.014	0.980–1.049	.426
HOMA‐IR	1.124	0.973–1.297	.112
QUICKI	0.743	0.564–0.981	**.036**
HbA1c (%)	1.116	0.782–1.594	.545
Prediabetes + Diabetes mellitus	1.621	0.528–4.973	.398
Metabolic syndrome	12.564	3.098–50.952	**<.001**
MRI Liver FF (%)	1.037	0.990–1.085	.126
MRI Pancreas FF (%)	1.205	1.039–1.398	**.014**

*Note*: Odds ratios of WHtR and QUICK were odds ratios for hypertension by every 0.01 increment of WHtR and QUICKI.

*Abbreviations*: ALT, alanine aminotransferase; AST, aspartate aminotransferase; BMI z‐score, body mass index standard deviation score; CI, confidence interval; FF, fat fraction.; FFMI, fat‐free mass index; FMI, fat free mass index; GGT, γ‐glutamyl transferase; HbA1c, glycosylated hemoglobin; HDL‐C, high density lipoprotein cholesterol; HOMA‐IR, homeostatic model assessment for insulin resistance; LDL‐C, low density lipoprotein cholesterol; MRI, magnetic resonance imaging; OR, odds ratio; QUICKI, quantitative insulin‐sensitivity check index; WHtR, waist circumference to height ratio.

### 3.4 Multiple logistic regression analysis for hypertension in children with NAFLD

Multiple logistic regression analysis was performed after selecting noncollinear covariates through the stepwise selection method to predict the risk factors associated with HTN. The remaining significant variables were BMI‐z and PFF. For the selection of variables to be adjusted in the multiple logistic regression, we examined the multicollinearity among the variables. Because children and adolescents are growing, the z‐score form is more appropriate than the anthropometric values. As BMI‐z implies a concept for adjusting for sex and age, it is reasonable that sex and age were not included in the multiple logistic regression model to avoid multicollinearity. Likewise, the criteria of MetS definition includes HTN. Therefore, the MetS status was not included as an adjusted variable. Because the WC‐z adjusted for age and sex has not yet been developed, we used WHtR as a substitute for WC‐z. However, WHtR was eliminated due to a high multicollinearity with BMI‐z (correlation matrix for the parameter estimates output 0.79). Likewise, as FMI and FFMI were relative to each other, a high multicollinearity between FMI and FFMI was shown (correlation matrix for the parameter estimates output 0.82). Because the *p*‐value of FFMI was more significant than that of FMI, we chose FFMI. Finally, we selected six variables for the multiple logistic analysis: BMI‐z, PFF, FFMI, GGT, dyslipidemia, and QUICKI.

Both BMI‐z (OR 4.912; 95% confidence interval [CI], 1.463–16.497; *p* = .010) and PFF (OR 1.279; 95% CI, 1.007–1.624; *p* = .044) were independent risk factors for HTN after adjusting for FFMI, GGT, dyslipidemia status, and QUICKI in children with NAFLD (Table 3). Particularly, MRI‐measured PFF was significantly associated with HTN independently of BMI‐z in the fully adjusted model (OR 1.279, 95% CI, 1.007–1.624, *p* = .044).

**TABLE 3:** Multiple logistic regression analysis for predicting risk factors associated with hypertension in 65 children with non‐alcoholic fatty liver disease

	Simple logistic regression		Multiple logistic regression	
	OR (95% CI)	*p*‐value	OR (95% CI)	*p*‐value
BMI z‐score	3.971 (1.842–8.563)	**<.001**	4.912 (1.463–16.497)	**.010**
FFMI	1.444 (1.141–1.828)	**.002**	0.926 (0.664–1.290)	.649
GGT	1.021 (1.004–1.038)	**.016**	1.007 (0.980–1.035)	.632
Dyslipidemia	5.357 (1.100–26.089)	**.038**	2.180 (0.212–22.441)	.512
QUICKI	0.743 (0.564–0.981)	**.036**	0.917 (0.588–1.430)	.702
MRI pancreas FF (%)	1.205 (1.039–1.398)	**.014**	1.279 (1.007–1.624)	**.044**

*Note*: Odds ratio of QUICK were odds ratio for hypertension by every 0.01 increment of QUICKI.

*Abbreviations*: BMI‐z, body mass index standard deviation score; CI, confidence interval; FF, fat fraction; FFMI, fat free mass index = FFM(Kg)/(height[m])^2^; GGT, γ‐glutamyl transferase; HOMA‐IR, homeostatic model assessment for insulin resistance = fasting plasma glucose (mg/dl) × fasting insulin (μU/ml)/405; MRI, magnetic resonance imaging; OR, odds ratio; QUICKI, quantitative insulin‐sensitivity check index = 1/log (HOMA‐IR × 405).

### 3.5 Optimal cut‐off value of PFF and BMI‐z for hypertension in children with NAFLD

**FIGURE 1:**
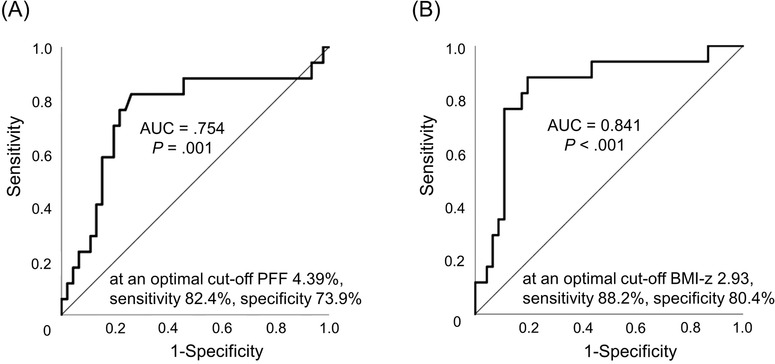
Optimal cut‐off value for PFF and BMI‐z for hypertension in children with NAFLD. Receiver operating characteristic (ROC) curves were constructed to examine the optimal cut‐off value for pancreatic fat fraction (PFF) and body mass index standard deviation score (BMI‐z) to divide children with non‐alcoholic fatty liver disease into two groups, with and without hypertension. The area under the ROC curve (AUC) was 0.754 (95% confidence interval [CI], 0.629–0.854, *p* = .001) at a cut‐off PFF of 4.39%, a sensitivity of 82.4%, a specificity of 73.9%, a positive predictive value of 53.9%, and a negative predictive value of 91.9% (A). The AUC was 0.841 (95% CI, 0.728–0.921, *p* < .001 at a cut‐off BMI‐z of 2.93, a sensitivity of 88.2%, a specificity of 80.4%, a positive predictive value of 62.5%, and a negative predictive value of 94.9% (B). However, BMI‐z was not superior to PFF in the prediction of hypertension when a pairwise comparison of the ROC curves was performed to confirm diagnostic superiority (difference between areas 0.0876, 95% CI, ‐0.100–0.275, *p* = .360)

The ROC curve was constructed to determine the optimal cut‐off value for PFF to divide the patients into two groups: those with and those without HTN. The area under the ROC curve (AUC) was 0.754 (95% CI 0.629–0.854, *p* = .001) at a cut‐off PFF of 4.39%, a sensitivity of 82.4%, a specificity of 73.9%, a positive predictive value of 53.9%, and a negative predictive value of 91.9%) (Figure 1).

The ROC curve was also constructed to determine the optimal cut‐off value for BMI‐z to divide the patients into two groups: those with and without HTN. The AUC was 0.841 (95% CI, 0.728–0.921, *p* < .001 at a cut‐off BMI‐z of 2.93, a sensitivity of 88.2%, a specificity of 80.4%, a positive predictive value of 62.5%, and a negative predictive value of 94.9%) (Figure 1).

When a pairwise comparison of the ROC curves was performed to confirm diagnostic superiority, BMI‐z was not superior to PFF for dividing the patients into two groups: those with and without HTN (difference between areas 0.0876, 95% CI, ‐0.100–0.275, *p* = .360). Therefore, PFF was not inferior to BMI‐z in the diagnosis of HTN. MRI‐measured PFF performed similarly in predicting HTN compared with BMI‐z. PFF had high values for predicting patients with HTN as much as BMI‐z.

## 4 DISCUSSION

To our knowledge, this is the first study to investigate the risk factors for developing HTN using MRI‐measured HFF, PFF, and obesity‐related metabolic components in children with liver biopsy‐proven NAFLD. In our study, multiple logistic regression analysis showed that BMI‐z and PFF were independent risk factors for HTN in children with biopsy‐proven NAFLD after adjusting for FFMI, GGT, dyslipidemia, and QUICKI, which were associated with HTN through simple logistic regression analysis. Especially, MRI‐measured PFF was significantly associated with HTN in the fully adjusted model, including BMI‐z. Furthermore, PFF was not inferior to BMI‐z in the suggestion of HTN when confirmed through a pairwise comparison of the ROC curves of BMI‐z and PFF.

NAFLD is known to be a strong cardiovascular risk factor, independent of traditional cardiovascular risk factors including obesity.^14^ A systematic review and meta‐analysis incorporating almost 165 000 participants in 34 studies showed the association of NAFLD with both prevalent and incident CVD, including coronary artery disease, atherosclerosis, and HTN.^31^ Furthermore, increasing scientific studies on children with NAFLD suggest that NAFLD in children as well as in adults might be an independent risk factor for CVD.^6^, ^7^, ^9^, ^11^ The recent clinical practice guidelines for the management of NAFLD in children recommend screening of the cardiovascular system for all patients with NAFLD.^6^, ^10^ At least a detailed risk factor evaluation and regular monitoring is recommended.^6^, ^10^

Possible mechanisms leading to CVD in patients with NAFLD might originate from the expanded and inflamed visceral fat.^5^ NASH may play a part in the pathogenesis of CVD through the systemic release of several inflammatory, hemostatic, and oxidative‐stress mediators, or through the contribution of NAFLD to IR and atherogenic dyslipidemia.^5^, ^14^

IR is a shared pathologic condition supporting several dysmetabolic status of obesity including prediabetes/type 2 DM, dyslipidemia, atherosclerosis, and NAFLD.^32^ In children and adolescents with obesity, a strong association between IR and a higher prevalence of MetS components has been observed; thus, a higher cardiovascular risk is predicted in these patients.^32^ IR has been demonstrated to be a reliable marker in the prediction of cardiovascular risk.^32^ It has been suggested that IR may be involved in the pathogenesis of atherosclerosis, according to the evidence that the more IR is increased in youths, the more circulating biomarkers of endothelial dysfunction are elevated, while adiponectin, which plays an antiatherogenic role, is reduced.^32^ The relevant association between IR and cardiovascular risk in the pediatric population is well known, especially in obese children,^32^ even though further research is needed to clarify this association.

In our study, QUICKI, which is indicative of IR, was significantly associated with HTN in a simple logistic regression analysis, but not with HOMA‐IR. We were not able to clearly explain why QUICKI, but not HOMA‐IR, was significantly associated with HTN. The gold standard for the assessment of IR was the hyperinsulinemic‐euglycemic clamp study; however, its costs and difficult management in clinical and research practice have determined the need of surrogate markers.^32^ It is known that HOMA‐IR and QUICKI present a favorable correlation with the hyperinsulinemic‐euglycemic clamp,^32^ and display identical diagnostic accuracy.^33^ Even though a previous study insisted that HOMA‐IR was more reliable than QUICKI as a measure of IR among children and adolescents,^34^ there were some studies showing that QUICKI displayed better reproducibility than HOMA‐IR.^35^, ^36^ Moreover, in hypertensive obese patients, QUICKI was well‐correlated with BP and with brain natriuretic peptide deficiency.^37^ A relative natriuretic peptide deficiency, probably related to the IR found in obese individuals, may represent one of the reasons for inducing HTN by IR.^32^

As it is presumed that IR may be associated with ectopic fat accumulation in the pancreas, we hypothesized that pancreatic steatosis may also affect the development of CVD through IR and undiscovered mechanisms. According to a meta‐analysis on pancreatic ectopic fat, non‐alcoholic fatty pancreas disease was associated with a significantly increased risk of arterial HTN (risk ratio 1.67, 95% CI, 1.32–2.10, *p* < .0001).^17^ Several studies have shown that MRI‐evaluated PFF was highly correlated with HTN.^38^, ^39^ One previous study also showed that fatty pancreas is a contributing factor for the development of atherosclerosis in adults with biopsy‐proven NAFLD.^40^

The present study additionally revealed that FFMI was significantly associated with HTN. A British study demonstrated that body fat was significantly higher and skeletal muscle mass was significantly lower in any subcategory of HTN in both men and women; thus, the estimation of both body fat and skeletal muscle mass may be important in clinical approach.^41^ In another adult study, patients with sarcopenic obesity had a greater risk of HTN than those with simple obesity or sarcopenia alone.^42^ According to a pediatric study on healthy Chinese children, a high fat‐free mass percentage was associated with a low BP and low HTN risk in the fully adjusted model.^43^

In our study, the GGT level was associated with the development of HTN. Longitudinal studies that assessed the association between NAFLD and the incidence of HTN in adults showed that GGT levels were significantly related to the incidence of HTN.^44^ Moreover, a study on children with NAFLD confirmed that GGT might be a potentially reliable, simple, and non‐invasive biochemical marker for the estimation of cardiovascular risk in obese children with NAFLD.^6^, ^45^

Dyslipidemia is known to increase the risk of developing HTN after adjusting for age, BMI, DM, alcohol, smoking, exercise, and parental history of HTN.^46^ The mechanisms through which obesity causes HTN are complex, including sympathetic nervous system over‐activation, stimulation of the renin‐angiotensin‐aldosterone system, alterations in adipose‐derived cytokines, structural functional renal changes, and IR.^47^ Therefore, more studies are needed to prove the association between PFF and HTN in the future.

Our study included 18 (27.7%) patients with non‐obese NAFLD. Of these, 13 (20.0%) were overweight (85^th^ ≤ BMI < 95^th^ percentile) and five (7.7%) were within the normal range (BMI < 85^th^ percentile). A systematic meta‐analysis showed the prevalence of NAFLD in children and adolescents with normal weight to be 2.3% (95% CI, 1.5%–3.6%), in overweight patients to be 12.5% (95% CI, 9.2%–16.7%), and in obese patients to be 36.1% (95% CI, 24.6%–49.4%) in studies conducted among the general population.^3^ However, the prevalence of non‐obese NAFLD in adults is higher than that in children. When the difference in the BMI cut‐off value to determine obesity of adults (cut‐off of BMI < 30 kg/m^2^ from the West and BMI < 25 kg/m^2^ from the East) was considered, epidemiological data indicated that the prevalence of non‐obese NAFLD in adults was 10%–30% (7%–21% for Western studies and 3%–27% for Eastern studies).^48^ Non‐obese NAFLD patients tend to be younger, male, and have lower BP, FPG, HbA1c levels, and genetic polymorphisms compared with obese NAFLD patients.^49^ Compared with obese patients without NAFLD, non‐obese NAFLD patients were found to have similar HOMA‐IR, HbA1c, and dyslipidemia.^49^

The present study had some limitations. First, this study did not recruit healthy children or overweight/obese children without NAFLD as controls. Although the comparison with age‐ and sex‐matched healthy children as normal controls would be ideal, this was not possible because of ethical considerations regarding subjecting healthy children to undergo MRI and blood sampling. In addition, overweight/obese children without NAFLD were not included because of ethical considerations. It would be inappropriate to perform abdominal MRI merely to measure ectopic fat content. Second, the visceral adipose tissue (VAT) or subcutaneous adipose tissue (SAT) content was not measured because of limitations in the software used in the present study. VAT plays an essential role in the development of IR and may serve as a missing link between IR and fatty pancreas.^12^, ^50^ SAT is recognized as a defense mechanism to prevent ectopic fat accumulation and, therefore, prevents metabolic degeneration and dysfunction of β‐cells.^12^, ^50^ Third, as this was a retrospective study with a relatively small sample size, we caution against over‐interpreting the significance of the results. In our center, liver biopsy and abdominal MRI are performed in patients at increased risk for severe liver disease. These tests are performed when persistently elevated liver enzymes and associated dyslipidemia, type 2 DM, and HTN are observed. This suggests that the results of the present study may not be generalizable to patients with mild NAFLD and/or no metabolic risk factors. However, it is critical to identify which pediatric patients with NAFLD are at greatest risk for cardiometabolic dysregulation, including HTN. Providing active intervention early to prevent disease progression is essential. Lastly, although we found an association between PFF and HTN, we did not analyze the data of carotid intima‐media thickness or carotid‐femoral pulse wave velocity to reflect atherosclerosis and pro‐inflammatory adipokines as a link between PFF and HTN, because these values were not measured in all study patients.

In conclusions, in addition to BMI‐z, ectopic pancreatic fat deposition is an important risk factor for developing HTN in children with NAFLD. Furthermore, PFF was not inferior to BMI‐z in suggesting patients with HTN. This study shows the importance and necessity of interpreting MRI‐measured PFF as well as HFF in conjunction with anthropometric and laboratory findings of obesity in assessing pediatric patients suspected of NAFLD. Measuring PFF as well as HFF on MRI could help predict the development of HTN in children with NAFLD. It would be possible to give early preventive measures to these young people to halt the development of HTN. Further large‐scale prospective studies are warranted to compare patients with NAFLD with age‐ and sex‐matched normal controls or overweight/obese children without NAFLD. Future studies should analyze VAT and SAT together with HFF and PFF to confirm the association between HFF, PFF, and metabolic components. More research is needed in the future to study the association between PFF and atherosclerosis parameters in children with NAFLD to confirm PFF as a risk factor for HTN.

## ACKNOWLEGMENTS

The authors thank the Division of Statistics in Medical Research Collaborating Center at Seoul National University Bundang Hospital for assisting in statistical analysis. This study was supported by the 2019 Research grant of the Korean Pediatric Society (Seokcheon Research Award).

## CONFLICT OF INTEREST

The authors have no potential conflicts of interest to disclose.

## AUTHOR CONTRIBUTIONS

Conceptualization: Hye Ran Yang, Eun Hye Lee, Data curation: Eun Hye Lee, Ji Young Kim, Formal analysis: Eun Hye Lee, Hye Ran Yang, Methodology: Hye Ran Yang, Ji Young Kim, Investigation: Eun Hye Lee, Ji Young Kimm, Supervision: Hye Ran Yang, Writing‐original draft: Eun Hye Lee, Writing‐review & editing: Eun Hye Lee, Hye Ran Yang. All authors read and approved the final manuscript.
